# Risk factors for *Blastocystis* infection in HIV/AIDS patients with highly active antiretroviral therapy in Southwest China

**DOI:** 10.1186/s40249-019-0596-7

**Published:** 2019-10-17

**Authors:** Shun-Xian Zhang, Fen-Yan Kang, Jia-Xu Chen, Li-Guang Tian, Lan-Lan Geng

**Affiliations:** 1 Guangzhou Institute of Pediatrics, Guangzhou Women and Children’s Medical Center, Guangzhou Medical University, Guangzhou, 510623 People’s Republic of China; 2 Department of Gastroenterology, Guangzhou Women and Children’s Medical Center, Guangzhou Medical University, Guangzhou, 510623 People’s Republic of China; 3 The Gansu Center for Disease Control and Prevention, Lanzhou, 730000 People’s Republic of China; 4 National Institute of Parasitic Diseases, Chinese Center for Disease Control and Prevention, Shanghai, 200025 People’s Republic of China; 5 Key Laboratory for Parasitology and Vector Biology, MOH of China, WHO Collaborating Center for Tropical Diseases, National Center for International Research on Tropical Diseases, Shanghai, 20025 People’s Republic of China

**Keywords:** *Blastocystis*, HIV/AIDS, Co-infection, Risk factor, Interaction

## Abstract

**Background:**

*Blastocystis* is a widespread zoonotic protozoan of mammalian species, especially in HIV/AIDS individuals. The aim of this study was to analyze the prevalence and risk factors related with *Blastocystis* infection among HIV/AIDS patients in Southwest China.

**Methods:**

The cross-sectional study was performed in 311 HIV/AIDS cases in Tengchong City, Yunnan Province from July 2016 to March 2017. For each subject, stool specimen was collected to detect the *Blastocystis*, and the blood sample was used to detect HIV virus load and CD4^+^ T cell count, in addition, structured questionnaire was used to collect the basic information and risk factors.

**Findings:**

The result showed that the detection rate of *Blastocystis* was 3.86% (95% *CI*: 2.22–6.62) among HIV/AIDS patients. Both raising animal (*OR* = 12.93, 95% *CI*: 1.54–108.36) and drinking un-boiled water (*OR* = 8.17, 95% *CI*: 1.76–37.90) were risk factors for *Blastocystis* infection in HIV/AIDS individuals. In addition, the interaction of CD4^+^ T cell count and HIV virus load was also contribution to *Blastocystis* infection (*P* = 0.007).

**Conclusions:**

A high prevalence of *Blastocystis* infection was found in HIV/AIDS patients in Tengchong. Poor hygienic habits, the interaction of HIV virus load and CD4^+^ T cell count were identified as main risk factors for infection. These results will help us to develop efficient control strategies to intervene with and prevent the occurrence of *Blastocystis* among HIV-infected individuals.

## Multilingual abstracts

Please see Additional file 1 for translations of the abstract into the five official working languages of the United Nations.

## Background

Despite the expansion of antiretroviral treatment programme several years ago, 940 000 people died from AIDS related illnesses and 1.8 million people became newly infected with HIV/AIDS, it remains a global public health problem [1–4]. Currently, dramatic expansion of the pandemic has brought about a significant change in the prevalent of pathogens all over the world, especially in developing countries [5–7]. HIV/AIDS and many intestinal pathogen, including *Cryptosporidium parvum*, *Blastocystis* [8–10], previously were considered to be sporadic or zoonotic infection, becoming opportunistic infection for individual.

*Blastocystis* is a single-cell, anaerobic eukaryotic organism [11, 12]. It is one of the most frequently intestinal parasite that found in human beings and other animals in the worldwide [13]. And about 1 billion people in the worldwide were infected by *Blastocystis* with ubiquitous asymptomatic infection [11, 12]. The detection rate of *Blastocystis* was 0.5–57.0% in developed countries [14, 15], and 30.0–60.0% in developing countries, especially in tropical, subtropical and poorly sanitized countries or regions [12]. Most importantly, the presence of the *Blastocystis* has been well documented among HIV/AIDS patients [5, 6, 16]. Some studies have reported that the prevalence was 0.8–2.2% in HIV-infected individuals [6, 17], whereas the detection of *Blastocystis* was 16.2% in HIV-infected patients conducted in China [5]. Taken together, these findings suggested that the prevalence of the *Blastocystis* among HIV/AIDS patients was variation in different regions of the world, is essential to further our knowledge of the epidemiology and clinical relevance of this organism in HIV-positive patients.

However, no available reports about the risk factors of *Blastocystis* infection among HIV-infected patients in Southwest China. This cross-sectional study was conducted to explore the risk factors affecting *Blastocystis* infection among HIV/AIDS, providing strategies for *Blastocystis* prevention and treatment.

## Methods

### Study design and subjects

From 1st July 2016 to 31st March 2017, this cross-sectional study was conducted in the Tengchong City, Yunan Province, China. A total number of 2279 HIV-infected patients were registered in Tengchong Center for Disease Control and Prevention, these patients were received standardized treatment, such as the highly active antiretroviral therapy (HAART), in the People’s Hospital of Tengchong City and the Tengchong Center for Disease Control and Prevention.

The participants in this study were randomly selected. The inclusion criteria for selection of participants involved who over 5 years old and is able to give written informed consent or to obtain assent by legal guardians, and is absence of obvious severe defects of development or malignant diseases affecting investigation procedures, while inadequate fecal sample, incomplete questionnaire, and refusal to participate were ruled out.

### Sample size calculation

The sample size was determined using the formula for sample size calculation [7].

$\mathrm{n}=\frac{z_{\alpha}^2p\left(1-p\right)}{\delta^2}$, *α* = 0.05, Z_0.05_ = 1.96, *δ* = precision of the event of interest = 0.05, where *n* = sample size, *p* = prevalence of *Blastocystis* among HIV/AIDS patients = 16.23% [5]. A minimum size was 209 cases, considering the 10% loss follow up, the final minimum size was 209 (1 + 0.1) = 230 participants. Finally, 311 HIV/AIDS patients were enrolled into this study.

### Questionnaire survey

One standardized structural questionnaire was designed to obtain socioeconomic and demographic description about each HIV/AIDS patient, including the age, gender, height, weight, education, residence, marital status, occupation and presence of symptoms, total family members and minor members in family, HIV infection time, route and medical treatments. In addition, environmental conditions, such as water supply, drinking water, toilet type and presence of domestic animals, were also included. This work were performed by trained doctors or nurses.

### Stool collection

Each fecal specimen was collected with sterile container and delivered to the laboratory of the People’s Hospital of Tengchong City, and stored at − 70 °C.

### Blood collection

Two milliliter venous blood of the aseptic processing procedures from each subject were collected with heparinized biomedical polymer anticoagulative tube, and transported to the laboratory of the People’s Hospital of Tengchong City immediately.

### Laboratory testing

#### Stool DNA extraction

Total genomic DNA was extracted with the QIAmp DNA Stool Mini Kit (Qiagen, Hilden, Germany) from stool specimen according to the manufacturer’s recommended procedures. Finally, genomic DNA was obtained and stored at − 70 °C until use.

#### Molecular detection of Blastocystis

Polymerase chain reaction (PCR) amplification was conducted to detect *Blastocystis* using the primers, targeted at the 18 ribosomal small subunitribosomal ribonucleic acid (SSU rRNA) coding region gene [18]. The forward primer was 5′-GGAGGTAGTGACAATAAATC-3′, and the reverse primer was 5′- ACTAGGAATTCCTCGTTCATG-3′, and the length of the PCR amplification product was 1100 bp [18], all primers were synthesized by Sangon Biotech Company (Shanghai, China). The PCR reaction mixture (25 μl total volume) consisted of 12.5 μl 2 × TaKaRa *Taq*™ mixture (TaKaRa Bio Inc., Shiga, Japan), 2 μl genomic DNA template, 1 μl each of 10 μmol/L forward primer and reverse primer, and 8.5 μl water. The PCR conditions consisted of one denaturing cycle at 94 °C for 5 min, 40 cycles involving denaturation at 94 °C for 30 s, annealing at 53 °C for 1 min, and extending at 72 °C for 1 min, followed by 72 °C for 10 min. The PCR product was subjected to 1% agarose gels at 120 V for 40 min and observed under UV light. The PCR product of suspected positive case was sent to purify and sequence using the dideoxy-terminal method by the Applied Biosystems 3130 Genetic Analyzer (Applied Biosystems, Foster City, California, USA). The result was compared with known sequences listed in the GenBank database maintained by the US National Library of Medicine (http://www.ncbi.nlm.nih.gov/BLAST/), using the basic local alignment search tool (Blast).

#### Analysis of CD4^+^ T cell counts

Blood sample was centrifuged at 1000×*g* for 10 min, and the supernatant (serum) was carefully collected, aliquoted in RNase-free EP tubes. The peripheral blood mononuclear cells were obtained from the precipitation of the whole blood and suspended in phosphate buffer solution (PBS) followed by adding antibodies of anti-human CD11a labeled FITC and PE conjugated anti-human CD4 (BD Biosciences, Franklin Lakes, New Jersey, USA). After incubation at 4 °C for 10 min, cells were suspended and centrifuged at 1000×*g* for 10 min again to remove the supernatant. The cells were suspended in 0.5 ml PBS and analyzed by BD FACS Count System (BD Biosciences, Franklin Lakes, New Jersey, USA). Negative control was set to determine the cut-off value.

#### Detection of HIV virus load

Then HIV virus load in the serum was determined with NucliSens HIV-1 QT Amplification Kit (BioMerieux, Marcyl’Etoile, France) using a virus load detector NucliSENS ECL (BioMerieux, Marcyl’Etoile, France) following the manufacturer’s instruction. The copy number of viral nucleic acid were measured to represent viral genome titers.

### Data analysis

The database was generated with EpiData 3.1 software (The EpiData Association, Odense, Denmark), and all data were recorded with double individuals and tested for consistency. Statistical analysis was performed with the IBM SPSS Statistics 25.0 software package (International Business Machines Corporation, Armonk, New York, United States). Odds ratio (*OR*) and 95% confidence interval (*CI*) of categorical variables were calculated using two tailed, Chi-square or Fisher’s exact test. Quantitative variable was described as mean, median, standard deviation or inter-quartile range (IQR), quantitative variable was compared by rank-sum test, analysis of variance or *t* test, significant difference was considered as the level of *P* <  0.05 with two-tailed test. The variables with *P* <  0.20 in the univariate analysis were introduced in the multivariate logistic regression analysis. The stepwise regression method was used. The proposed standard was *P* > 0.20, the final test level was *P* <  0.05 with two-tailed.

## Results

### Basic information and clinical symptoms of subjects

A total number of 311 HIV patients, including 149 male and 162 female, were recruited in our study from 1st July 2016 to 31st March 2017. The average age, weight, and height were 40 years (95% *CI*: 39–41), 57 kilogramme (95% *CI*: 56–58) and 162 cm (95% *CI*: 161–162), respectively. While the average number of family individual and juvenile were 4 (95% *CI*: 4–5) and 1 (95% *CI*: 1–1), respectively.

Among 311 HIV/AIDS patients, in the grade of education, the person with junior middle school-level education was most, followed by primary school-level education, high school-level education and university or college-level education. Based on mode of transmission, sexual transmission was predominant transmission route, followed by syringe transmission and mother to child transmission. What’s more, the average CD4^+^ T cell count and HIV virus load were 520 cells/μl (95% *CI*: 495–544) and 2587 copies/ml (95% *CI*: 315–4859), respectively. The average treatment time was 68 months (95% *CI*: 64–72) for all patients.

In addition, the most common clinical symptoms were loss of appetence (19.3, 95% *CI*: 15.3–24.0), followed by skin itching (17.0, 95% *CI*: 13.3–21.6), abdominal distension (16.1, 95% *CI*: 12.4–20.6), pruritus (13.5, 95% *CI*: 10.1–17.8), abdominal pain (12.9, 95% *CI*: 9.6–17.0), anemia (2.9, 95% *CI*: 1.5–5.4) and chronic diarrhea (1.0, 95% *CI*: 0.3–2.8).

### *Blastocystis* prevalence and the relationship between *Blastocystis* infection and clinical symptom

**Fig. 1:**
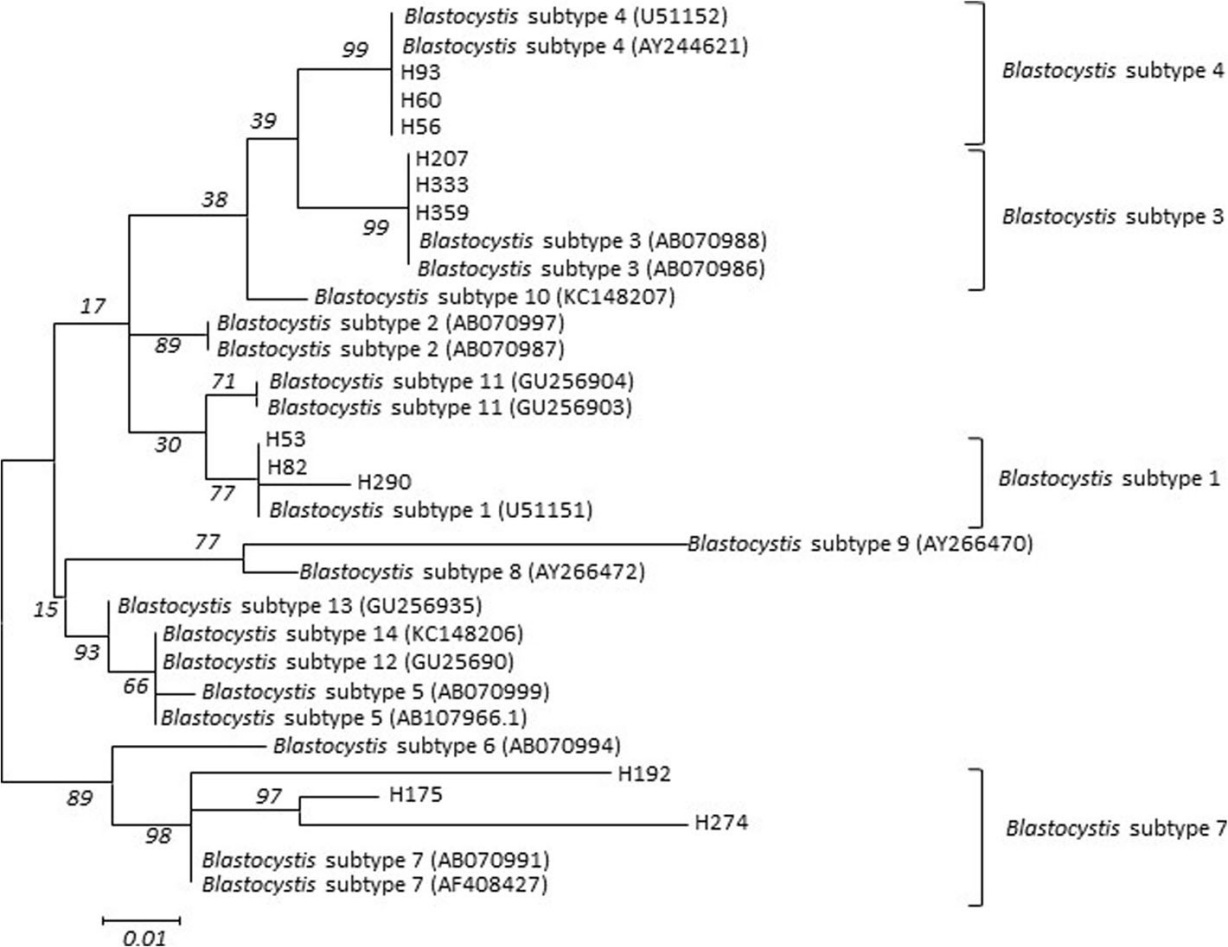
Generation of evolutionary tree of *Blastocystis* with neighbor-joining analysis. The reference sequence was obtained from GeneBank. 12 cases were diagnosed as *Blastocystis* infection, *Blastocystis* subtype 1, subtype 3, subtype 4 and subtype 7 were three, identically

Twelve cases of 311 HIV/AIDS subjects were infected with *Blastocystis*, and the detection rate was 3.86% (95% *CI*: 2.22–6.82) (Fig. 1, Additional file 2). No significant association was observed between *Blastocystis* infection and clinical symptoms, such as diarrhea (*P* = 0.999), abdominal distension (*P* = 0.999), loss of appetence (*P* = 0.060), itchy skin (*P* = 0.437), perianal pruritus (*P* = 0.063) and anemia (*P* = 0.320).

### Risk factors for the *Blastocystis* infection with univariate analysis

Univariate analysis has revealed that drinking water, raising livestock, HIV infection route, CD4^+^ T cell count and HIV virus load were closely association with *Blastocystis* infection (Table 1). In addition, the potential risks (*P* <  0.20) for the *Blastocystis* infection were gender and washing hand after defecation (Table 1). Whereas, several factors have no influence on *Blastocystis* infection among HIV-infected patients (Table 1), such as age, nationality, residence, education level, marriage, family member, body mass index (BMI), water source, toilet type, keeping pet, household member chronic diarrhea and HIV clinical stage.

**Table 1:** Single factor analysis of influencing factors for *Blastocystis* infection among HIV patients

Variable		*Blastocystis* (+) *n =* 12 *N* (%)	*Blastocystis* (−) *n =* 299 *N* (%)	Univariate analysis		
				χ^2^	*P* value	*OR* (95% *CI*)
Age	< 40 year (*n =* 179)	8 (4.5)	171 (95.5)	0.424	0.515	0.67 (0.20–2.27)
	≥ 40 year (*n =* 132)	4 (3.0)	128 (97.0)			
Gender	Male (*n =* 149)	3 (2.0)	146 (5.6)	2.656	0.105	2.86 (0.76–10.78)
	Female (*n =* 162)	9 (98.0)	153 (94.6)			
Nationality	Minority nationality (*n =* 11)	1 (9.1)	11 (90.9)	–	0.356	0.38 (0.04–3.24)
	Han nationality (*n =* 300)	10 (3.7)	289 (96.3)			
Residence	Urban (*n =* 260)	10 (3.8)	250 (96.2)	0.001	0.980	1.02 (0.22–4.80)
	Rural area (*n =* 51)	2 (3.9)	49 (96.1)			
Education level	Primary school (*n =* 132)	6 (4.5)	126 (95.5)	1.583	0.633	–
	Junior middle school (*n =* 161)	6 (3.7)	155 (96.3)			
	High school (*n =* 15)	0 (0.0)	15 (100.0)			
	University or collage (*n =* 3)	0 (0.0)	3 (100.0)			
Marriage	Unmarried (*n =* 25)	0 (0.0)	25 (100.0)	4.487	0.213	–
	Married (*n =* 259)	12 (4.6)	247 (95.4)			
	Married and living alone or widowed (*n =* 22)	0 (0.0)	22 (100.0)			
	Other (*n =* 5)	0 (0.0)	5 (100.0)			
Family member	< 5 individuals (*n =* 48)	1 (2.1)	47 (97.9)	–	0.701	2.05 (0.26–16.27)
	*≥* 5 individuals (*n =* 263)	11 (42)	252 (95.8)			
Body mass index	Underweight (*n =* 29)	2 (6.9)	27 (93.1)	2.862	0.210	–
	Normal (*n =* 271)	9 (3.3)	262 (96.7)			
	Overweight (*n =* 11)	1 (9.1)	10 (90.7)			
Drinking water	Boiled water (*n =* 291)	7 (2.4)	284 (97.6)	–	< 0.001	13.50 (3.80–47.70)
	Un-boiled water (*n =* 20)	5 (25.0)	15 (75.0)			
Water source	No-tap water (*n =* 11)	0 (0.0)	11 (100.0)	–	0.999	–
	Tap water (*n =* 300)	12 (4.0)	288 (96.0)			
Toilet type	Water wash toilet (*n =* 135)	3 (2.2)	132 (97.8)	1.722	0.789	2.37 (0.63–8.93)
	Un-water wash toilet (*n =* 176)	9 (5.1)	167 (94.9)			
Washing hand after defecation	No (*n =* 12)	2 (16.7)	10 (3.3)	–	0.071	0.17 (0.03–0.90)
	Yes (*n =* 299)	10 (3.3)	289 (96.7)			
Keeping pet	No (*n =* 230)	8 (3.5)	222 (96.5)	–	0.518	1.44 (0.44–4.92)
	Yes (*n =* 81)	4 (4.9)	77 (95.1)			
Raising animal	No (*n =* 146)	1 (0.7)	145 (99.3)	–	0.006	10.36 (1.32–81.23)
	Yes (*n =* 165)	11 (6.7)	154 (93.3)			
HIV infection route	Syringe (*n =* 18)	3 (16.7)	15 (83.3)	6.239	0.044	–
	Mother to children (*n =* 6)	0 (0.0)	6 (100.00)			
	Sex (*n =* 287)	9 (3.1)	278 (96.9)			
Take antiviral drug	No (*n =* 2)	0 (0.0)	2 (100.0)	–	0.999	1.04 (1.02–10.06)
	Yes (*n =* 309)	12 (3.9)	297 (96.1)			
Household member chronic diarrhea	No (*n =* 294)	10 (3.4)	2 (11.8)	–	0.134	3.79 (0.76–18.84)
	Yes (*n =* 17)	2 (11.8)	15 (88.2)			
CD4^+^ T cell count	< 500 (*n =* 139)	1 (0.6)	171 (99.4)	11.141	0.001	0.07 (0.01–0.53)
	*≥* 500 (*n =* 172)	10 (3.6)	268 (96.4)			
HIV virus load	< 50 (*n =* 282)	7 (2.5)	275 (97.5)	–	0.002	8.18 (2.41–27.75)
	*≥* 50 (*n =* 29)	5 (17.2)	24 (82.8)			
HIV clinical stage	I stage (*n =* 138)	7 (5.1)	131 (94.9)	2.439	0.486	–
	II stage (*n =* 73)	3 (4.1)	70 (95.9)			
	III stage (*n =* 82)	2 (2.4)	80 (97.6)			
	IV stage (*n =* 18)	0 (0.0)	18 (100.0)			

The “–” symbol indicates the data was not be calculated

*OR* Odd ratio, *CI* Confidence interval

### Risk factors for the *Blastocystis* infection with multivariate analysis

Based on these variables (drinking water, raising livestock, HIV infection route, CD4^+^ T cell count, HIV virus load, gender and washing hand after defecation) were involved in the multivariate model, further analysis showed that only four factors were association with *Blastocystis* infection as follows: raising animal, drinking water, CD4^+^ T cell count and HIV virus load (Table 2).

**Table 2:** Multivariate logistic regression analysis of influencing factors for *Blastocystis* infection among HIV patients

Variable	B	SE	Wald	df	*P* value	*OR* (95% *CI*)
Raising livestock	2.548	1.082	5.54	1	0.019	12.78 (1.53–106.63)
Drinking water	2.109	0.781	7.286	1	0.007	8.24 (1.78–38.12)
CD4^+^ T count	2.377	1.088	4.773	1	0.029	10.75 (1.28–90.90)
HIV virus load	1.769	0.764	5.365	1	0.021	5.86 (1.31–26.19)
Constant	−9.208	2.886	10.184	1	< 0.001	–
Dummy variable was defined and entered in multivariate logistic regression model						
Raising animal	2.559	1.085	5.568	1	0.018	12.93 (1.54–108.36)
Drinking water	2.100	0.783	7.195	1	0.007	8.17 (1.76–37.90)
CD4^+^ T*HIV			12.199	3	0.007	–
CD4^+^ T*HIV(1)	−4.034	1.203	11.248	1	0.001	0.02 (0.00–0.19)
CD4^+^ T*HIV(2)	−19.829	14 457.616	< 0.001	1	0.999	–
CD4^+^ T*HIV(3)	−1.869	0.793	5.554	1	0.018	0.15 (0.03–0.73)
Constant	−7.990	2.267	12.422	1	< 0.001	–

The “–” symbol indicates the data was not be calculated

*B* Beta, *SE* Standard error, *OR* Odd ratio, *CI* Confidence interval

### Interaction effect among CD4^+^ T cell count and HIV virus load for *Blastocystis* infection

Upon the threshold of CD4^+^ T cell count and HIV virus load was set into 500 cells/μl and 50 copies/ml, respectively. The detection rate of *Blastocystis* was different in these four groups (Table 3). At the same time, the interaction variable between the HIV virus load and CD4^+^ T cell count, named CD4^+^ T*HIV, which was introduced in Table 3. As for the new variable CD4^+^ T*HIV (Table 3), the group 4 was defined as reference group (dummy variable), and analysis was performed again, including the factor that drinking water, raising animal, HIV infection route, CD4^+^ T cell count, HIV virus load, gender and washing hand after defecation. The furher results showed that raising animal and drinking un-boiled water were the risk factors for *Blastocystis* infection among HIV/AIDS cases, and the new variable CD4^+^ T*HIV was also contribution the *Blastocystis* prevalence (Table 2). In addition, the detection rate of *Blastocystis* in subjects with HIV virus load < 50 copies/ml and CD4^+^ T <  500 cells/μl was less than that in individuals with HIV virus load ≥50 copies/ml and CD4^+^ T <  500 cells/μl (*OR* = 0.02, 95% *CI*: 0.00–0.19), and the prevalence of *Blastocystis* in subjects with HIV virus load ≥50 copies/ml and CD4^+^ T ≥ 500 cells/μl was lower than that in individuals with HIV virus load ≥50 copies/ml and CD4^+^ T < 500 cells/μl (*OR* = 0.15, 95% *CI*: 0.03–0.73) (Table 3).

**Table 3:** Effect of HIV virus load and CD4^+^ T cell count on *Blastocystis* infection among HIV patients

Group	*Blastocystis* (+) *N*	*Blastocystis* (−) *N*	Total *N*	Detection rate (%, 95 *CI*)	Group
HIV virus load < 50 copies/ml	7	275	282	2.48 (1.21–5.03)	–
HIV virus load *≥*50 copies/ml	5	24	29	17.20 (7.60–34.55)	–
CD4^+^ T < 500 cells/μl	11	128	139	7.91 (4.47–13.61)	–
CD4^+^ T *≥* 500 cells/μl	1	171	172	0.58 (0.10–3.22)	–
HIV virus load < 50 copies/ml and CD4^+^ T < 500 cells/μl	6	111	117	5.13 (2.37–10.12)	1
HIV virus load < 50 copies/ml and CD4^+^ T *≥* 500 cells/μl	1	164	165	0.61 (0.11–3.36)	2
HIV virus load *≥*50 copies/ml and CD4^+^ T *≥* 500 cells/μl	0	7	7	0.00 (0.00–35.43)	3
HIV virus load *≥*50 copies/ml and CD4^+^ T < 500 cells/μl	5	17	22	22.73 (10.74–43.44)	4 (Reference)

The “–” symbol indicates the data was not be calculated

## Discussion

*Blastocystis* is one of the most common enteric protozoa in HIV-infected patient due to weaken immunity [19]. In this study, the detection rate of *Blastocystis* was 3.70% in HIV-infected patients, it was significant lower than that reported by others conducted in HIV/AIDS patients in China [5], and some developing countries, such as Ethiopia (10.6%) [20] and Iran (19.0%) [21]. Conversely, the detection rate of *Blastocystis* was higher than that in non-diarrhea subjects in China (32.6%) [22]. However, the prevalence of *Blastocystis* in this study was closely to other studies conducted among non-diarrhea and non-HIV population (4.0%) in urban area in China [23, 24], it may be attributed to the subject enrolled in this study, once HIV/AIDS patient was found in China, the large dose antiviral drug was used to treat, resulting in the low HIV virus load in serum, at the same time, the high immune status of patients can prevent intestinal protozoa infection to some extent, in addition, some broad-spectrum antibiotics were used to prevent opportunistic infection in the processes of the standardized treatment for HIV/AIDS patient.

In line with other study [22], the result also showed that drinking un-boiled water was risk factor for *Blastocystis* infection among HIV/AIDS patients, it may increase the infection chance for intestinal protozoa, especially in HIV/AIDS patients. What’s more, raising animal was another risk factor, it was consist with the report by Wang et al. showed that the HIV/AIDS patients could be infected by frequently contacting with livestock infected with *Blastocystis* [25]. Hence, the economic condition, raising livestock, and lifestyle remain to be improved, it is important event in blocking the infection of the *Blastocystis* and reducing the *Blastocystis* prevalence.

In this study, the average number of CD4^+^ T cell count was 453 cells/μl in HIV cases infected with *Blastocystis*, it was lower than that of in healthy people (> 500 cells/μl). A study by Fekadu et al. showed that CD4^+^ T cell count will be degradation among HIV/AIDS patient [26]. Implying weaken immunity caused by low CD4^+^ T cell count may contribute *Blastocystis* infection in HIV/AIDS patients, and it was reasonable that the HIV/AIDS cases should be receive standardized treatment and long-term monitoring [21]. However, other study have showed that low CD4^+^ T cell count was not major risk factor for *Blastocystis* infection [27], for instance, no significant differences of *Blastocystis* infection was observed in HIV/AIDS individuals with or without CD4^+^ T cell count more than 200 cells/μl [7], and another study showed that compared to HIV/AIDS patients with CD4^+^ T cell count less than 50 cells/μl, patients with CD4^+^ T cell count more than 50 cells/μl were not more likely to be infected by *Blastocystis* [28]. In addition, another study have also suggested that high HIV virus load was risk factor for intestinal protozoa infection [29], while another study found that the HIV concentration has no effect on the enteric parasites infection [6]. Interestingly, our study revealed that the prevalence of *Blastocystis* in HIV/AIDS cases with high HIV virus load and low CD4^+^ T cell count was much higher than that in other groups, implying that *Blastocystis* infection among HIV/AIDS subjects was not only association with HIV virus load and CD4^+^ T cell count, but also depended on the interaction effect between these two variables. These findings suggested that CD4^+^ T cell count have inversely correlated with HIV virus load, both of them are the risk factors of *Blastocystis* infection among HIV/AIDS subjects.

There were several shortcomings in this study needed to be addressed. It was a cross-sectional study and cannot be obtained causal conclusion. At the same time, data sparsity issue led to be fail to estimate for risk factors of *Blastocystis* infection among HIV/AIDS cases. Hence, the sample size should be expanded to explore the interaction effect between HIV virus load and CD4^+^ T cell count during *Blastocystis* infection in future.

## Conclusions

Both raising animal and drinking un-boiled water were risk factors for *Blastocystis* infection, and the interaction of CD4^+^ T cell count and HIV virus load was also contribution to *Blastocystis* infection. Thus, improvement of health education, good hygiene and living habit are important to prevent and control *Blastocystis* infection. In addition, HIV-infected individuals must be treated by with HAART, it could be effect to reduce the HIV virus load and prevent *Blastocystis* infection.

## Supplementary information

**Additional file 1.** Multilingual abstracts in the five official working languages of the United Nations.

**Additional file 2.** The sequence of *Blastocystis* in this study.

## Data Availability

Data of the study can be available upon request from the author (LG-T).

## Abbreviations

AIDS: Acquired immune deficiency syndrome
Blast: Basic local alignment search tool
BMI: Body mass index
*CI*: Confidence interval
DNA: Deoxyribonucleic acid
HAART: Highly active antiretroviral therapy
HIV: Human immunodeficiency virus
IQR: Inter-quartile range
*OR*: Odd ratios
PBS: Phosphate buffer solution
PCR: Polymerase chain reaction
RNA: Ribonucleic acid
SSU rRNA: Ribosomal small subunit, ribosomal ribonucleic acid

## References

[1] Hemelaar J, Elangovan R, Yun J, Dickson-Tetteh L, Fleminger I, Kirtley S, Williams B (2019). Global and regional molecular epidemiology of HIV-1, 1990-2015: a systematic review, global survey, and trend analysis. Lancet Infect Dis.

[2] Shan D, Yu MH, Yang J, Zhuang MH, Ning Z, Liu H, Liu L (2018). Correlates of HIV infection among transgender women in two Chinese cities. Infect Dis Poverty..

[3] Ji YJ, Liang PP, Shen JY, Sun JJ, Yang JY, Chen J, Qi TK (2018). Risk factors affecting the mortality of HIV-infected patients with pulmonary tuberculosis in the cART era: a retrospective cohort study in China. Infect Dis Poverty.

[4] Gibson RM, Nickel G, Crawford M, Kyeyune F, Venner C, Nankya I, Nabulime E (2017). Sensitive detection of HIV-1 resistance to zidovudine and impact on treatment outcomes in low- to middle-income countries. Infect Dis Poverty..

[5] Tian LG, Chen JX, Wang TP, Cheng GJ, Steinmann P, Wang FF, Cai YC (2012). Co-infection of HIV and intestinal parasites in rural area of China. Parasit Vectors.

[6] Hosseinipour MC, Napravnik S, Joaki G, Gama S, Mbeye N, Banda B, Martinson F (2007). HIV and parasitic infection and the effect of treatment among adult outpatients in Malawi. J Infect Dis.

[7] Nsagha DS, Njunda AL, Assob NJC, Ayima CW, Tanue EA, Kibu OD, Kwenti TE (2016). Intestinal parasitic infections in relation to CD4(+) T cell counts and diarrhea in HIV/AIDS patients with or without antiretroviral therapy in Cameroon. BMC Infect Dis.

[8] Tan KS (2008). New insights on classification, identification, and clinical relevance of *Blastocystis* spp. Clin Microbiol Rev.

[9] Stark D, Barratt JL, van Hal S, Marriott D, Harkness J, Ellis JT (2009). Clinical significance of enteric protozoa in the immunosuppressed human population. Clin Microbiol Rev.

[10] Hunter PR, Nichols G (2002). Epidemiology and clinical features of *Cryptosporidium* infection in immunocompromised patients. Clin Microbiol Rev.

[11] Wang J, Gong B, Liu X, Zhao W, Bu T, Zhang W, Liu A, Yang F (2018). Distribution and genetic diversity of *Blastocystis* subtypes in various mammal and bird species in northeastern China. Parasit Vectors.

[12] Udonsom R, Prasertbun R, Mahittikorn A, Mori H, Changbunjong T, Komalamisra C, Pintong AR (2018). *Blastocystis* infection and subtype distribution in humans, cattle, goats, and pigs in central and western Thailand. Infect Genet Evol.

[13] Ramírez JD, Sánchez A, Hernández C, Flórez C, Bernal MC, Giraldo JC, Reyes P (2016). Geographic distribution of human *Blastocystis* subtypes in South America. Infect Genet Evol.

[14] Dogan N, Aydin M, Tuzemen NU, Dinleyici EC, Oguz I, Dogruman-Al F (2017). Subtype distribution of *Blastocystis* spp. isolated from children in Eskisehir, Turkey. Parasitol Int.

[15] Fletcher S, Caprarelli G, Merif J, Andresen D, Hal SV, Stark D, Ellis J (2014). Epidemiology and geographical distribution of enteric protozoan infections in Sydney, Australia. J Public Health Res.

[16] Tian LG, Wang TP, Lv S, Wang FF, Guo J, Yin XM, Cai YC (2013). HIV and intestinal parasite co-infections among a Chinese population: an immunological profile. Infect Dis Poverty..

[17] Ghimire A, Bhandari S, Tandukar S, Amatya J, Bhandari D, Sherchand JB (2016). Enteric parasitic infection among HIV-infected patients visiting Tribhuvan University teaching hospital, Nepal. BMC Res Notes.

[18] Wong KH, Ng GC, Lin RT, Yoshikawa H, Taylor MB, Tan KS (2008). Predominance of subtype 3 among *Blastocystis* isolates from a major hospital in Singapore. Parasitol Res.

[19] Adarvishi S, Asadi M, Cheshmeh MGD, Tavalla M, Hardani F (2016). Prevalence of intestinal parasites in HIV-positive patients attending Ahvaz health centers in 2012: a cross-sectional study in south of Iran. Jundishapur J Chronic Dis Care.

[20] Alemu A, Shiferaw Y, Getnet G, Yalew A, Addis Z (2011). Opportunistic and other intestinal parasites among HIV/AIDS patients attending Gambi higher clinic in Bahir Dar city, north West Ethiopia. Asian Pac J Trop Med.

[21] Piranshahi AR, Tavalla M, Khademvatan S (2018). Genomic analysis of *Blastocystis* hominis isolates in patients with HIV-positive using locus SSU-rDNA. J Parasit Dis.

[22] Li LH, Zhou XN, Du ZW, Wang XZ, Wang LB, Jiang JY, Yoshikawa H (2007). Molecular epidemiology of human *Blastocystis* in a village in Yunnan province. China Parasitol Int.

[23] Zhang SX, Yang CL, Gu WP, Ai L, Serrano E, Yang P, Zhou X (2016). Case-control study of diarrheal disease etiology in individuals over 5 years in Southwest China. Gut Pathog.

[24] Zhang SX, Zhou YM, Xu W, Tian LG, Chen JX, Chen SH, Dang ZS (2016). Impact of co-infections with enteric pathogens on children suffering from acute diarrhea in Southwest China. Infect Dis Poverty..

[25] Wang W, Owen H, Traub RJ, Cuttell L, Inpankaew T (2014). Bielefeldt-Ohmann H3. Molecular epidemiology of *Blastocystis* in pigs and their in-contact humans in Southeast Queensland, Australia, and Cambodia. Vet Parasitol.

[26] Fekadu S, Taye K, Teshome W, Asnake S (2013). Prevalence of parasitic infections in HIV-positive patients in southern Ethiopia: a cross-sectional study. J Infect Dev Ctries.

[27] Cardoso LV, Galisteu KJ, Schiesari Júnior A, Chahla LA, Canille RM, Belloto MV, Franco C (2011). Enteric parasites in HIV-1/AIDS infected patients from a northwestern Sao Paulo reference unit in the highly active antiretroviral therapy era. Rev Soc Bras Med Trop.

[28] Paboriboune P, Phoumindr N, Borel E, Sourinphoumy K, Phaxayaseng S, Luangkhot E, Sengphilom B (2014). Intestinal parasitic infections in HIV-infected patients. Lao People's Democratic Republic PLoS One.

[29] Roka M, Goñi P, Rubio E, Clavel A (2013). Intestinal parasites in HIV-seropositive patients in the continental region of Equatorial Guinea: its relation with socio-demographic, health and immune systems factors. Trans R Soc Trop Med Hyg.

