# Supplementary material for: Risk factors for Blastocystis infection in HIV/AIDS patients with highly active antiretroviral therapy in Southwest China
Source: Infect Dis Poverty. 2019 Oct 17;8:89. doi: 10.1186/s40249-019-0596-7 (PMC6796344; doi:10.1186/s40249-019-0596-7)
Supplement: Supplementary file 1 — Additional file 1. Multilingual abstracts in the five official working languages of the United Nations. [file 40249_2019_596_MOESM1_ESM.pdf]

Translation of the abstract into the five official working languages of the United Nations

العوامل المعرضة لخطر الإصابة بعدوى المتبرعمة الكيسية لدى مرضى العوز المناعي المكتسب الخاضعين للعلاج المتعدد ذي الفعالية العالية ضد فيروسات النسخ العكسي في جنوب غرب الصين

شون شيان تشانغ، فان يان قانغ، جيا شو تشن، لي قوانغ تيان، لان لان قنغ

ملخص

السياق: تعد المتبرعمة الكيسية إحدى الأوالي الحيوانية ذات الانتشار الواسع بين الثدييات وخاصة لدى المصابين بنقص المناعة. كان الهدف من هذه الدراسة هو تحليل نسبة الإصابة بالمتبرعمة الكيسية والعوامل المعرضة لحدوثها بين مرضى العوز المناعي في جنوب غرب الصين.

الأساليب: تم إجراء دراسة شاملة على 311 حالة من العوز المناعي في مدينة تنغتشونغ بمقاطعة يونان من يوليو 2016 إلى مارس 2017. تم جمع عينة من البراز لكل حالة بهدف الكشف عن المتبرعمة الكيسية بينما تم استخدام عينة الدم لقياس حمولة فيروس نقص المناعة وعدد الخلايا التائية المساعدة، كما تم كذلك استخدام استبيان منسق من أجل جمع المعلومات الأساسية وعوامل الخطر المحتملة.

النتائج: أظهرت النتيجة أن معدل اكتشاف المتبرعمة الكيسية كان 3.86% (95% *CI*: 2.22–6.62) بين مرضى نقص المناعة. حيث كان كل من تربية الحيوان (*OR* = 12.93، 95% *CI*: 1.54–108.36) وشرب الماء غير المغلي (*OR* = 8.17، 95% *CI*: 1.76–37.90) من العوامل المعرضة للإصابة بعدوى المتبرعمة الكيسية لدى المصابين بنقص المناعة. كما كان التأثير المشترك لتعداد الخلايا التائية المساعدة ونسبة حمولة فيروس نقص المناعة عاملا مساهما في حدوث الإصابة بعدوى المتبرعمة الكيسية (*P* = 0.007).

الاستنتاجات: تمت ملاحظة نسبة عالية من الإصابة بعدوى المتبرعمة الكيسية لدى مرضى العوز المناعي. إذ تم تحديد العادات الصحية غير السليمة إلى جانب التأثير المشترك لنسبة حمولة فيروس نقص المناعة وتعداد الخلايا التائية المساعدة كعوامل رئيسية لاحتمال حدوث الإصابة. ستساعدنا هذه النتائج على وضع استراتيجيات فعالة للمكافحة من أجل منع الإصابة بالمتبرعمة الكيسية والتدخل عند حدوثها بين الأفراد المصابين بنقص المناعة.

Translated from English version into Arabic by Aghilas Mihoub, revised by Amine Abdoune, through

TRANSLATORS WITHOUT BORDERS

中国西南地区高效抗逆转录病毒治疗的 HIV/AIDS 人群中人芽囊原虫感染情况及危险因素研究

张顺先，康芬艳，陈家旭，田利光，耿岚岚

摘要

引言: 人芽囊原虫是哺乳动物中广泛流行的一种自然疫源性病原体，尤其流行于 HIV/AIDS 人群。本研究旨在揭示中国西南地区 HIV/AIDS 人群中人芽囊原虫的流行程度及危险因素。

方法: 2016 年 7 月到 2017 年 3 月期间，我们在中国云南省腾冲市开展了该项横断面研究，总共 311 名 HIV/AIDS 研究对象被招募到本研究，对每一位研究就对象，我们无菌采集粪便以便检查人芽囊原虫，采集血液检测 HIV 病毒载量和 $CD4^+$ T 含量，同时，我们使用结构化问卷收集研究对象的基本信息和相关的危险因素。

结果: 311 名 HIV/AIDS 研究对象中，人芽囊原虫的检出率为 3.86%（95% *CI*: 2.22–6.62）。饲养动物（*OR* = 12.93，95% *CI*: 1.54–108.36）和饮用未煮沸的水（*OR* = 8.17，95% *CI*:1.76–37.90）是 HIV/AIDS 人群感染人芽囊原虫的危险因素，最关键的是低 CD4+T 细胞含量与高 HIV 病毒载量之间存在相互作用，它们相互促进，并与人芽囊原虫的感染存在正向关系（*P* = 0.007）。

结论：人芽囊原虫的感染率在 HIV/AIDS 人群不可忽视。不良的卫生习惯、较高的 HIV 病毒载量和较低的 $CD4^+$ T 细胞数量是影响人芽囊原虫感染的危险因素。本研究的结果将提高我们对人芽囊虫流行程度的认知，有助于我们制定有效的控制策略，以干预和预防 HIV/AIDS 人群人芽囊原虫的感染和流行。

Translated from English version into Chinese by Shun-Xian Zhang

Facteurs de risque de l'infection à *Blastocystis* chez les patient.e.s atteint.e.s. du SIDA/VIH sous thérapie antirétrovirale hautement active dans le sud-ouest de la Chine.

Shun-Xian Zhang, Fen-Yan Kang, Jia-Xu Chen, Li-Guang Tian, Lan-Lan Geng

Résumé

Contexte: *le Blastocystis* est un protozoaire zoonotique répandu des espèces mammifères, présent particulièrement chez les individus atteints du SIDA/VIH. Le but de cette étude était d'analyser la prévalence et les facteurs de risque liés à l'infection à *Blastocystis* chez les patient.e.s atteint.e.s du VIH/SIDA dans le sud-ouest de la Chine.

Méthodes: L'étude-transversale a été effectuée sur 311 cas de SIDA/VIH à Tengchong, dans la province du Yunnan, entre juillet 2016 et mars 2017. Pour chaque sujet, des échantillons de selles ont été prélevés afin de détecter le *Blastocystis*, et le prélèvement sanguin a servi à détecter la charge virale du VIH ainsi que le nombre de cellules $CD4^{+\,T}$; un questionnaire structuré a servi à recueillir les informations et les facteurs de risque de base.

Constatations: Le résultat a montré que le taux de détection du *Blastocystis* était de 3,86 % (95% *CI*: 2,22–6,62) chez les patient.e.s. atteint.e.s. du VIH/SIDA. Aussi bien l'élevage d'animaux (*OR* = 12,93, 95% *CI*: 1,54–108,36) que la consommation-d'eau non bouillie (*OR* = 8,17, 95% *CI*: 1,76–37,90) constituaient des facteurs de risque de l'infection à *Blastocystis* chez les individus atteints du VIH/SIDA. De plus, l'interaction entre le nombre de cellules $CD4^{+\,T}$ et la charge virale du VIH ont aussi contribué à l'infection à *Blastocystis* (*P* = 0,007).

Conclusions: Une forte prévalence de l'infection à *blastocystis* a été constatée chez les patient.e.s. atteint.e.s. du VIH/SIDA. De mauvaises habitudes d'hygiène, ainsi que l'interaction entre la charge virale du VIH et le nombre de cellules $CD4^{+\,T}$ ont été identifiés comme les principaux facteurs de risques d'infection. Ces résultats nous aideront à développer des stratégies de contrôle efficaces afin de combattre et de prévenir l'occurrence du *Blastocystis* parmi les individus atteints du VIH.

Translated from English version into French by Bettina Stefani, revised by Lisa Séguélas, through

TRANSLATORS WITHOUT BORDERS

Факторы риска заражения *бластоцистозом* больных ВИЧ/СПИД при высокоактивной антиретровирусной терапии на юго-западе Китая

Шунь-Сянь Чжан, Фэнь-Янь Кан, Цзя-Сюй Чэнь, Ли-Гуан Тянь, Лань-Лань Гэн

Аннотация

Справочная информация: *Бластоцисты*являются широко распространенными зоонозными простейшими среди млекопитающих видов, особенно у людей с ВИЧ/СПИДом. Цель этого исследования – проанализировать распространенность и факторы риска инфицирования *бластоцистозом* среди пациентов с ВИЧ/СПИДом на юго-западе Китая.

Методы: С июля 2016 года по март 2017 года было проведено перекрестное исследование среди 311 зараженных ВИЧ/СПИДом в городе Тэнчун, провинция Юньнань. В каждом случаебыл взят образец стула, чтобы определить наличие *бластоцист*, и анализ крови для обнаружения вирусной нагрузки ВИЧ и количества Т-клеток $CD4^+$, в дополнение к этому использовалась стандартизированная анкета для сбора основной информации и факторов риска.

Заключение: Результаты показали, что частота выявления *бластоцистоза* равнялась 3.86% (95% *ДИ*: 2.22–6.62) среди больных ВИЧ/СПИДом. Разведение животных (*ОШ* = 12.93, 95% *ДИ*: 1.54–108.36) и употребление не-кипяченой воды (*ОШ* = 8.17, 95% *ДИ*: 1.76–37.90) были факторами риска заражения *бластоцистозом* для людей с ВИЧ/СПИДом. Помимо этого, взаимоотношение Т-клеток $CD4^+$ и вирусной нагрузки ВИЧ также способствовало заражению *бластоцистозом* (P = 0.007).

Выводы: Широкая распространенность *бластоцистоза* выявлена среди пациентов с ВИЧ/СПИДом. Было установлено, что низкий уровень гигиены, взаимоотношение вирусной нагрузки и количества Т-клеток $CD4^+$ являются основными факторами риска заражения. Эти результаты помогут нам разработать эффективные стратегии контроля для вмешательства и предотвращения возникновения *бластоцистоза* среди ВИЧ-инфицированных людей.

Translated from English version into Russian by Ekaterina Igosheva, revised by Anna Kukharchuk, through

TRANSLATORS
WITHOUT BORDERS

Factores de riesgo para la infección por *Blastocystis*, en pacientes con VIH/SIDA, que reciben un tratamiento antirretroviral altamente activo, en el suroeste de China

Shun-Xian Zhang, Fen-Yan Kang, Jia-Xu Chen, Li-Guang Tian, Lan-Lan Geng

Resumen

Contexto: *Blastocystis*es un organismo protozoario zoonótico de los mamíferos, que está muy extendido, especialmente en individuos con VIH/SIDA. El objetivo de este estudio consistió en analizar la prevalencia y los factores de riesgo relacionados con *la infección* por Blastocystis entre los pacientes con VIH/SIDA, en el suroeste de China.

Metodología: Se llevó a cabo un estudio transversal, con 311 casos de pacientes con VIH/SIDA, en la ciudad de Tengchong, provincia de Yunnan, desde julio de 2016 hasta marzo de 2017. Se recogió una muestra de heces, para cada sujeto, para detectar la presencia de *Blastocystis*, así como una muestra de

sangre, que se utilizó para detectar la concentración del virus del VIH y de linfocitos T $CD4^+$. Además, se utilizó un cuestionario, para recopilar la información básica y los factores de riesgo.

Resultados: Los resultados mostraron una tasa de detección de *Blastocystis* del 3,86% (*IC* 95%: 2,22–6,62), en pacientes con VIH/SIDA. Tanto la crianza de animales (*OR* = 12,93, *IC* 95%: 1,54–108,36) como el consumo de agua sin hervir (*OR* = 8,17, *IC* 95%: 1,76–37,90) se identificaron como factores de riesgo, para la infección por *Blastocystis*en individuos, con VIH/SIDA. Además, la interacción del recuento de linfocitos T $CD4^+$ y la concentración del virus VIH también contribuyó a la infección por *Blastocystis* (*P* = 0,007).

Conclusiones: Se encontró una prevalencia alta de infección por *Blastocystis* en pacientes con VIH/SIDA. Los malos hábitos de higiene, la interacción de concentración del virus VIH y de linfocitos T $CD4^+$ se identificaron como los principales factores de riesgo para la infección. Estos resultados nos ayudarán a desarrollar estrategias de control eficientes, con el fin de intervenir y prevenir la aparición de *Blastocystis*, en personas infectadas por VIH.

Translated from English version into Spanish by Noemí Jiménez, revised by María Luz Puerta, through

TRANSLATORS WITHOUT BORDERS
